# Cytotoxic activity of extracts and crude saponins from *Zanthoxylum armatum* DC. against human breast (MCF-7, MDA-MB-468) and colorectal (Caco-2) cancer cell lines

**DOI:** 10.1186/s12906-017-1882-1

**Published:** 2017-07-17

**Authors:** Fiaz Alam, Qazi Najum us Saqib, Abdul Waheed

**Affiliations:** 1 Department of Pharmacy, COMSATS Institute of Information Technology, Abbottabad, 22060 Pakistan; 2 Research Scientist Clinical Trials, Centre for Experimental Medicine & Rheumatology, Sir John Vane Science Centre, William Harvey Research Institute, Queen Mary University of London, Charterhouse Square, London, EC1M 6BQ UK

**Keywords:** *Zanthoxylum armatum*, Saponins, Breast cancer, Colorectal cancer, MTT, NRU, DAPI

## Abstract

**Background:**

*Zanthoxylum armatum* DC has been an important traditional plant known for its medicinal properties. It is well known for its antimicrobial, larvicidal and cytotoxic activities.

**Methods:**

The potential anticancer effects of the methanol extract and the crude saponins from fruit, bark and leaves of *Z. armatum* on breast (MDA-MB-468 and MCF-7) and colorectal (Caco-2) cancer cell lines using MTT, neutral red uptake(NRU) and DAPI stain assays were evaluated.

**Results:**

In MTT assay the methanol extract of fruit (Zf), bark (Zb) and leaves (Zl) of *Zanthoxylum armatum*, showed significant and dose dependent growth inhibition of MCF-7, MDA MB-468 and Caco-2 cancer cell lines in a dose of 200 μg/ml and above. The saponins (Zf.Sa, Zb.Sa and Zl.Sa) showed significant activity against MDA MB-468 (95, 94.5 and 85.3%) as compared to MCF-7 (79.8, 9.43, 49.08%) and Caco-2 (75.8, 61.8, 68.62%) respectively. The extracts were further tested in more sensitive NRU assay and its was found that Zf extract showed higher cytotoxic activity as compared to Zb and Zl extracts with 100 μg/ml concentration. The breast cancer cell lines showed more sensitivity toward the crude saponins from fruit and bark with maximum inhibition of up to 93.81(±2.32) % with respect to 71.19(± 2.76) of Actinomycin-D. DAPI staining experiment showed that saponins from fruit induced apoptosis mode of cell death in all three types of cell lines while saponins form leaves and bark showed similar results against MDA MB-468 indicated by nuclear fragmentation and chromatin condensation. The effect of saponins from fruit, bark and leaves (Zf.Sa, Zb.Sa and Zl.Sa) against Caco-2 cell lines inhibited the growth of Caco-2 by 53.16 (±3.31) %, 66.43 (± 3.24) and 45.96 (± 10.67) respectively with respect to Actinomycin-D (4 μM*)* which showed the growth inhibition of 65.40(±4.29) %.

**Conclusion:**

The current study clearly demonstrates that the extract and crude saponins from fruit, bark and leaves of traditional medicinal plant *Zanthoxyllum armatum* DC., has the potential to exert its cytotoxic effect on cancer cell lines isolated form human by a mechanism involving apoptosis. The overall finding demonstrate that this plant specially fruits, could be potential source of new anticancer compounds for possible drug development against cancer.

## Background

Cancer has become one of the most annihilating diseases globally with more than 10 million new cases annually. The compounds isolated from natural sources have been the source of most of the active components of medicines. One of the studies revealed that since 1994 50 % of the drugs approved are originated from natural sources. The drugs approved cover a variety of therapeutic indications, most of these are related to cancer treatment [1]. It is evident from various studies that medicinally important plants including *Podophyllum peltatum*, *Taxus brevifolia*, and *Cantharanthus roseus* and many others provided bioactive compounds including podophyllotoxin, taxol, and vincristine etc. for the management of various cancers [2]. There is always a need to search alternative sources with more effectiveness, more accessibility, and least side effects. Therefore, the present study was carried out to explore the anticancer activity of extracts and crude saponin fractions from *Zanthoxylum armatum* fruit, bark and leaves in cultured human breast cancer cells (MCF-7 and MDA MB-468) and colorectal cancer cell lines (Caco-2).

The genus Zanthoxylum have been explored for various activities based on traditional uses. The active metabolites isolated from these plants showed anti-cancer potential [3]. A similar study available from the same genus is about *Zanthoxylum usambarense* (Engl.). In this study the extracts of *Z. usambarense* showed significant activity against breast cancer cell lines MCF-7 (IC_50_ 42.9 mg/mL) and revealed to have induced cell death through apoptosis [4]. Another study showed that the plant *Zanthoxylum armatum* DC. extract has the ability to induce clumped chromosomes at metaphase stage of cell division coupled with mitotic arrest, DNA degradation, and chromatin condensation [3].

*Zanthoxylum armatum* (DC) belongs to family Rutaceae. It is a common plant in Southeast Asia. In Pakistan it is found wild in Dir, Hazara, and Murree hills of Pakistan [5]. The aerial parts of the plant are used in management of fever, dyspepsia, stomachic upset, cholera, and in toothache [6]. The small branches of *Z. armatum* are used as tooth brush (miswak) for washing the teeth while the powder of fruit is applied in toothache [7]. Many plants of *Zanthoxylum* genus possess antimicrobial, larvicidal and cytotoxic activity [8–11]. The different classes of chemical constituents reported from *Z. armatum* include terpenes, sterols, flavonoids, alkaloids, saponins, and coumarins [5, 12–14].

## Methods

### Chemicals

Neutral red solution, Fetal Bovine serum (FBS), Actinomycin-D, Dulbecco’s modified Eagle medium **(**DMEM) and 4, 6-diamidineo-2-phenylinldole (DAPI), MTT (methyl-thiazolyl tertrazolium), DMSO (dimthylsulphoxide) were obtained from Sigma Chemical Co. (St Lois, MO, USA). The drugs Gentamycin, Streptomycin, Glutamine, Actinomycin-D used in the experiments were also purchased from Sigma Chemical Co. (St Lois, MO, USA).

### Cell lines

MCF-7 and MDA-MB-468 cells were purchased from ATCC (American Type Culture Collection, USA) through an authorized distributor, LGC Standards, Teddington, UK. The Caco-2 (human colon adenocarcinoma) cell line was obtained from the ECCC (European Collection of Cell Cultures) through Health Protection Agency, Salisbury, UK (Catalogue No. 86010202).

### Plant material

Five kg of each of leaves, bark and fruit of *Z. armatum* were collected from Mansehra, Tanawal area of KPK Pakistan in the month of August, 2013. After authentication from taxonomist, the plant and its voucher specimen (PB025.13) were deposited in the herbarium of the Post graduate college, Abbottabad. Each part of the plant was washed under running water and dried in shade at room temperature and was ground to coarse powder. The powder drug was stored in air tight and light resistant container before extraction.

### Cell culture

A complete growth medium was provided for the cell growth: Dulbecco’s modified Eagle medium **(**DMEM) containing 10% *v*/v FBS, 2 mM L-glutamine, Gentamycin (40 μg/ml), Penicillin (100 units/ml) and Streptomycin (1040 μg/ml). The cells were seeded into 96-well cell culture plates at a density of 1 × 10^4^ cells per well in 100 μl aliquots of the medium. The cells were permitted to attach for a period of 24 h, the temperature was maintained at 37 °C, and 5% CO_2_ in air, in a humidified atmosphere in an incubator.

### Preparation of extract and crude saponins

The powder material (200 g each) of leaves, bark and fruit was extracted with methanol solvent using soxhlet extractor for 20 h. After filtering through Whatman Grade-I filter paper, the filtrate was evaporated under reduced pressure on a vacuum rotary evaporator at 40 °C. The separation of saponins from powdered materials (200 g) of leaves, bark and fruit was carried out first by defatting with petroleum ether. In the next step extraction was carried out with methanol in Soxhlet apparatus. Evaporation of the solvent was done under reduced pressure to obtain a semi solid extract. This extract of the plant was further fractionated with n-butanol and water in equal proportions. The n-butanol fraction was separated. Petroleum ether was added to the extract in small proportions to precipitate the crude saponins [15]. Extractive yield (percent) of the methanol extracts were; bark (Zb): 21 ± 1%, leaves (Zl):19 ± 1%, and fruit (Zf): 14.34 ± 1%.

### Preparation of the drug for the experiment

The crude extract of the leaf, bark and fruit of *Z. armatum* were tested for cell cytotoxicity against breast and colorectal cancer cell lines. A series of eight dilutions (10, 25, 50, 100, 200, 300, 400 and 500 μg of final concentration) of crude extracts were prepared in DMEM (100 μl) containing DMSO (Dimethyl sulfoxide, maximum: 0.01%). After the initial screening results, a test dose of 200 μg for MTT assay and 100 μg for NRU assay was set for extracts based on their apparent IC_50_ values.

### Cytotoxicity assays

After a 24 h exposure of test period, the determinations of toxic endpoints were carried out by two colorimetric methods namely; methyl-thiazolyl tertrazolium (MTT) assay and neutral red uptake (NRU) assay.

### MTT (methyl-thiazolyl tertrazolium) assay

The quantification of cancer cells growth was carried out as described by [16, 17]. After test period of 24 h exposure to extract, the cells were washed with phosphate buffer saline (PBS) twice. 10 μl of MTT reagent (5 mg/ml in PBS) was poured to each well including the blanks (contained medium only). The plates were incubated for 4 h at 37 °C. Afterward, cells were washed with PBS twice, and 100 μl/well DMSO was added as a solvent to dissolve the insoluble crystalline formazan products. The effect of plant leaves, bark and fruit extracts on cancer cells was quantified as the percentage of control absorbance of reduced dye at 550 nm on a microplate reader (LabtechLT-4000MS, Labtechm International Ltd., East Sussex, and UK). Five replicates wells were examined for each treatment, and each experiment was repeated three times (*n* = 3) to calculate the standard error of mean. The results were calculated as percentage growth inhibition, untreated (control) cells versus treated cells according to the following formula:

$$ \%\mathrm{Growth}\mathrm{Inhibition}\kern0.5em =\frac{\mathrm{Control}{\textstyle \hbox{-}}\mathrm{actualabcorbance}}{\mathrm{Control}}\times 100 $$

Absorbance of the media was subtracted, both from control and treated cells.

### NRU (neutral red uptake) assay

This assay was performed as described by [18]. The medium was removed after dosing cells. In the next step 200 μl of neutral red solution (40 μg/ml) was poured into each well of test solution and blank. The mixture was incubated for 2.5 h. and neutral red was removed. The cells were then rinsed with warm PBS carefully. 200 μl of ethanol/acetic acid (1% glacial acetic acid in 5% ethanol) was added to all wells. The 96 well plates were covered in foil. The plates were shacked for 30 min on shaker to separate neutral red dye from cells and to make the solution homogenous. The absorbance of the samples was measured within 60 min at 540 nm using a microplate reader. The samples were measured in replicates of five. Each experiment was performed three times (*n* = 3). The results were calculated by the same equation as already given for MTT assay.

### Cytomorphological alterations (DAPI staining)

In this study, DAPI (4′, 6-diamidino-2-phenylindole) stain was used to evaluate the morphological changes in nuclei of control and treated cells. The cells were seeded at density of 1 × 10^4^ cells/well in 500 μl of medium on sterilized glass cover slips in 96 well plates for 24 h. The cells were treated with the culture medium (negative control) and Actinomycin-D (positive control, 4 μM). The plates were incubated at 37 °C, 5% CO_2_ in air in a humidified atmosphere for 24 h. After treatment, cells were briefly equilibrated with PBS, fixed with 4% paraformaldehyde for 15 min, permeabilised with methanol for 5 min, and mounted in a DAPI-containing medium (Vector Shield, Vector Labs, Peterborough, UK). The morphology of the nuclei was observed using a confocal fluorescence microscope, Leica SP2 AOBS confocal microscope (Leica Microsystems, Mannheim, Germany) with excitation at 350 nm and emission 460 nm under a ×40 oil objective [19].

### Data presentation and statistical analysis

All data were compiled from a minimum of three experiments. Data for statistical analysis were expressed as ‘standard error of the mean’, n (number of experiments). The software used was GraphPad Prism version 6.00 for Windows, (GraphPad Software, San Diego California, USA). The test applied was Dunnett’s test with one-way ANOVA.

## Results

### MTT assay

The methanol extract and crude saponins from leaves, bark and fruit of *Zanthoxylum armatum* were subjected to MTT assay using two breast cancer cell lines, MDA-MB-468 and MCF-7, and the colorectal cell line Caco-2 to assess potential cytotoxicity.

The methanol extract fruit (Zf), bark (Zb) and leaves (Zl) of *Z. armatum*, showed a concentration dependent growth inhibition from 10 to 500 μg/ml of MCF-7 cancer cell lines. The Zf showed more effective inhibition and highly significant activity was observed with extract concentration of 200 μg/ml as compared to Zb (400 μg/ml) and Zl (300 μg/ml).

Similarly, a concentration depended response was observed against the growth of MDA MB-468 cell lines and very high significance inhibition was observed above the concentration of 200 μg/ml for Zf and Zb. However, Zl extract showed maximum inhibition over 300 μg/ml. The methanol extracts of fruit, bark and leaves of *Z. armatum* showed a concentration depended inhibition of Caco-2 cancer cell lines growth in tested concentration range of 10–500 μg/ml. The very high significance inhibition of the growth was observed above the concentration of 200 μg/ml for Zf and 300 μg/ml for Zb. However, Zl extract proved least active and showed maximum inhibition over 500 μg/ml. (Table 1).

**Table 1:** Cytotoxic activity of crude extract of Z*. armatum* using MTT assay with apparent IC_50_ about 200 μg/ml

Conc. (μg/ml)	*Z. armatum fruit extract (Zf)*			*Z. armatum leaves extract (Zl)*			*Z. armatum bark extract (Zb)*		
	MCF-7 cells	MDA MB-468 cells	Caco-2 cells	MCF-7 cells	MDA MB-468 cells	Caco-2 cells	MCF-7 cells	MDA MB-468 cells	Caco-2 cells
10	21.58* ±3.2	9.8 ± 3.6	15.96* ±6.8	3.56 ± 1.8	0.87 ± 0.4	3.83 ± 2.3	4.25 ± 3.8	16.51 ± 4.6	5.21 ± 1.4
25	37.50** ± 4.1	12.72* ±3.0	27.97* ±2.1	2.79 ± 1.0	2.50 ± 0.5	4.10 ± 2.1	6.08 ± 2.0	27.54* ±3.2	16.33 ± 1.2
50	35.45** ± 8.6	22.78* ±1.9	49.25 ** ± 5.0	11.22 ± 2.6	4.11 ± 0.9	8.60 ± 4.3	7.46 ± 5.5	42.25** ±3.4	12.63 ± 3.2
100	35.22** ± 5.1	38.90** ± 3.9	53.93*** ±4.0	26.14* ±5.0	6.23 ± 1.4	14.21 ± 2.0	11.18 ± 4.0	46.22** ±4.6	20.52* ±1.4
200	58.28*** ± 4.8	57.26*** ± 4.4	64.95*** ±4.2	35.71** ±4.7	20.97* ±2.3	21.05* ± 4.4	17.28 ± 3.9	74.39*** ±2.8	39.38** ±4.8
300	65.74*** ± 7.8	69.48*** ± 4.7	75.60*** ±8.5	54.35*** ± 4.6	41.97** ±1.1	28.56* ± 2.3	23.06* ± 3.2	74.87*** ±3.9	62.72*** ±3.0
400	73.70*** ± 10.2	85.15*** ± 2.8	88.90*** ±5.8	63.77*** ± 3.2	99.58*** ±0.2	41.63** ± 4.7	59.21*** ±2.9	84.24*** ±6.4	63.56*** ±4.7
500	95.88*** ± 6.0	90.54*** ±6.8	94.24*** ±4.3	78.63*** ± 4.2	99.35*** ±0.5	50.14*** ± 3.5	71.15*** ±2.6	99.76*** ±0.7	68.37*** ±2.8
Veh. Cont.	2.87 ± 0.6	4.92 ± 2.91	−2.10 ± 0.2	1.10 ± 1.13	1.57 ± 3.6	1.68 ± 5.2	1.69 ± 4.4	1.67 ± 1.0	1.76 ± 5.1

In the table statistically (Dunnett’s multiple comparison test) * = Significant (*P* < 0.05), ** = Highly significant (*P* < 0.01), *** = Very highly significant (*P* < 0.001)

The effect of saponins of *Z. armatum* fruit (Zf.Sa), bark (Zb.Sa) and Leaves (Zl.Sa) against cell lines MCF-7 and MDA MB-468 tested at a concentration of 100 μg/ml in MTT assays. The saponins Zf.Sa showed growth inhibition of MCF-7 and MDA-MB-468 by 79.89 (±7.45) % and 95 (±2.64) % respectively, the saponins Zb.Sa inhibited the growth by 9.43 (± 3.82) and 94.59 (± 3.00) and saponins of Zl.Sa inhibited with growth by 49.08 (± 5.21) and 85.33 (± 3.41) respectively with respect to Actinomycin-D (4 μM***)*** which showed the growth inhibition of 62.04 (±1.43) % and 62.87 (±5.28) % respectively. The effect of Zf.Sa, Zb.Sa and Zl.Sa against cancer cells Caco-2 were tested at dose of 200 μg/ml for MTT assays. The saponins showed maximum effect and inhibited the growth of Caco-2 by 75.88 (±8.41) %, 61.82 (± 4.07) and 68.62 (± 2.48) respectively with respect to Actinomycin-D (4 μM***)*** which showed the growth inhibition of 65.40(±4.29) % (Table 2).

**Table 2:** Cytotoxic activity of saponins fractions (200 μg/ml) from *Z. armatum* using MTT assay

Fractions (200 μg/ml)	Percentage growth inhibition of cancer cells (Mean ± Std. Dev.)			
	MCF-7 cells	MDA MB-468 cells	Caco-2 cells	Vehicle Control
*Zf.Sa*	79.69*** (±7.45)	95.00*** (±2.64)	75.88*** (±8.41)	3.17 (±5.67)
*Zl.Sa*	49.08** (±5.21)	85.33*** (±3.41)	68.62** (±2.48)	3.43 (±6.80)
*Zb.Sa*	9.43 (±3.82)	94.59*** (±3.00)	61.82** (±4.07)	0.20 (±2.34)
*Actinomycin-D (4* μM*)*	62.04** (±1.43)	62.87** (±5.28)	65.40** (±4.29)	1.10 (±1.13)

In the table statistically (Dunnett’s multiple comparison test) * = Significant (*P* < 0.05), ** = Highly significant (*P* < 0.01), *** = Very highly significant (*P* < 0.001)

### NRU assay

The *Z. armatum* Zf, Zb and Zl extracts showed the dose dependent growth inhibition of the MCF-7 and MDA MB-468 cancer cell lines from 10 to 500 μg/ml concentrations in NRU assay. Zf and Zb showed highly significance response with concentration of 100 μg/ml. Zl showed maximum response above the concentration of 300 μg/ml. The Zf, Zb and Zl showed the inhibition of the Caco-2 cancer cell lines and highly significance responses were observed with dose of 200 μg/ml for Zf and 400 μg/ml for Zb and Zl (Table 3).

**Table 3:** Cytotoxic activity of crude extract of *Z. armatum* using (NRU) neutral red uptake assay with apparent IC_50_ 100 μg/ml

Conc. (μg/ml)	*Z. armatum fruit saponins (Zf.Sa)*			*Z. armatum leaves saponins (Zl.Sa)*			*Z. armatum bark saponins (Zb.Sa)*		
	MCF-7 cells	MDA MB-468 cells	Caco-2 cells	MCF-7 cells	MDA MB-468 cells	Caco-2 cells	MCF-7 cells	MDA MB-468 cells	Caco-2 cells
10	16.02* ± 7.5	13.98 ± 3.9	25.76* ±2.1	2.71 ± 0.5	13.92 ± 3.6	5.74 ± 4.0	20.04* ±4.4	4.43 ± 1.4	3.64 ± 0.4
25	27.89* ±6.1	29.60* ±3.9	26.60* ± 4.2	3.07 ± 1.2	28.27* ± 3.2	7.42 ± 1.7	45.78** ±5.1	6.31 ± 1.2	4.46 ± 3.0
50	46.82** ±6.1	39.31** ± 3.9	30.52** ± 4.3	3.51 ± 1.4	25.76* ± 4.1	13.87 ± 5.0	66.05*** ±6.0	9.55 ± 0.9	4.03 ± 3.8
100	58.08*** ±7.1	53.53*** ±3.9	42.99** ± 3.3	4.27 ± 2.1	52.61** ± 5.6	10.18 ± 4.1	72.23*** ±3.2	28.91* ±5.0	19.59 ± 3.1
200	58.75*** ±4.0	55.75*** ±3.9	59.78*** ±6.7	34.39** ±2.9	77.43*** ± 5.4	32.90** ± 8.2	83.29*** ±3.6	99.36*** ±0.6	22.85 ± 5.1
300	71.62*** ±4.8	66.62*** ± 3.9	72.47*** ±5.6	93.73*** ±2.0	99.04*** ± 1.6	30.13** ± 2.5	92.55*** ±5.8	100.00*** ±0.2	30.98** ±8.4
400	80.89*** ±6.2	73.72*** ±3.9	91.78*** ±3.6	100.71*** ± .01	100.14*** ± 1.3	56.45*** ± 2.6	98.78*** ±1.2	100.00*** ±0.3	66.03*** ±5.4
500	86.18*** ±4.6	93.29*** ±3.9	96.86*** ±2.9	100.81*** ±0.2	100.14*** ± 0.5	63.97*** ± 3.4	99.08*** ±1.0	100.00*** ±0.6	90.01*** ±3.1
Veh. Cont.	−1.49 ± 1.0	1.38 ± 0.9	−3.45 ± 1.8	4.41 ± 2.2	1.91 ± 1.6	1.35 ± 3.7	1.72 ± 1.4	5.21 ± 3.0	−1.81 ± 0.9

In the table statistically (Dunnett’s multiple comparison test) * = Significant (*P* < 0.05), ** = Highly significant (*P* < 0.01), *** = Very highly significant (*P* < 0.001)

The effect of saponins of *Z. armatum* fruit (Zf.Sa), bark (Zb.Sa) and Leaves (Zl. Sa) against cancer cells MCF-7 and MDA MB-468 were tested at concentration of 100 μg/ml in NRU assays. The saponins Zf.Sa showed inhibition of MCF-7 and MDA MB-468 by 81.67 (±4.15) % and 93.81(±2.32) % respectively, the saponins Zb.Sa inhibited the growth by 7.77 (± 4.83) and 95.25 (± 4.35) and saponins of Zl. Sa inhibited with growth by 48.58 (±7.36) and 71.19(± 2.76) respectively with respect to Actinomycin-D (4 μM***)*** which showed the growth inhibition of 62.04 (±1.43) % and 62.87 (±5.28) % respectively. The crude saponins Zf.Sa, Zb.Sa and Zl.Sa inhibited the growth of Caco-2 by 53.16 (±3.31) %, 66.43 (± 3.24) % and 45.96 (± 10.67) % respectively with respect to Actinomycin-D (4 μM*)* which showed the growth inhibition of 65.40(±4.29) %. (Table 4).

**Table 4:** Cytotoxic activity of saponins fractions (100 μg/ml) from *Z. armatum* using NRU assay

Fractions (100 μg/ml)	Percentage growth inhibition of cancer cells (Mean ± Std. Dev.)			
	MCF-7 cells	MDA MB-468 cells	Caco-2 cells	Vehicle Control
*Zf.Sa*	81.67*** (±4.15)	93.81*** (±2.32)	53.16*** (±3.31)	−0.19 (±2.68)
*Zl.Sa*	48.58 (±7.36)	71.19 (±2.76)	45.96 (±10.67)	4.23 (±7.80)
*Zb.Sa*	7.77 (±4.83)	95.25***(±4.35)	66.43(±3.24)	2.92(±3.20)
*Actinomycin-D (4* μM*)*	91.84*** (±3.73)	93.94*** (±5.02)	65.97** (±4.83)	1.67 (±1.06)

In the table statistically (Dunnett’s multiple comparison test)* = Significant (*P* < 0.05), ** = Highly significant (*P* < 0.01), *** = Very highly significant (*P* < 0.001)

### DAPI staining

**Fig. 1:**
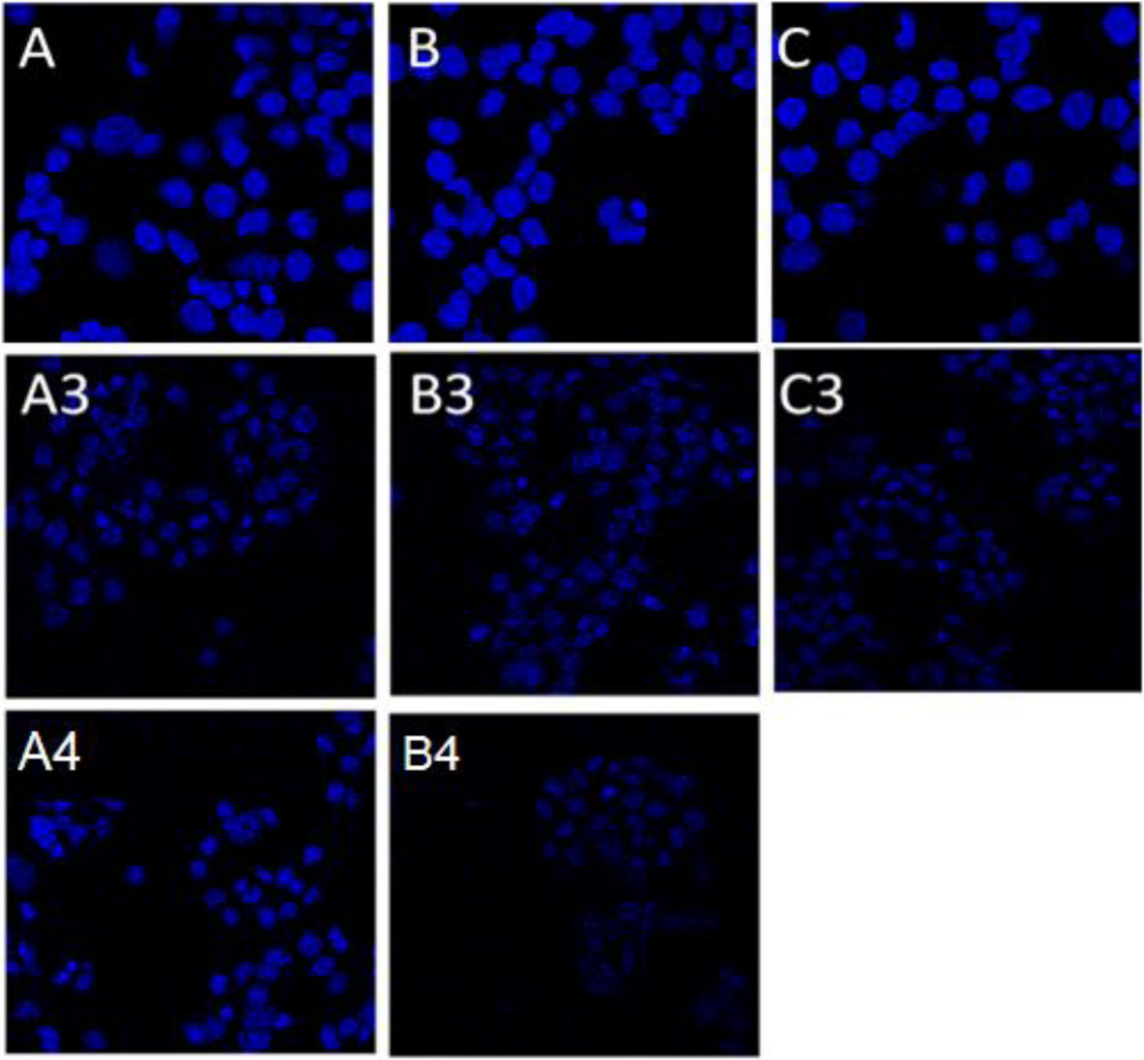
Cytmorphology of control cells (**a** = MCF-7, **b** = MDA MB-468, **c** = CaC02). Saponins from *Z. armatum* Zf treated cells (**a3**, **b3**, **c3**), Zb treated cells (**a4**), Zl treated cells (**b4**). (Cells were treated with most active saponins (10 μg/ml, final concentration) for 24 h and visualized under confocal microscope for DAPI stain)

The DAPI staining of cancer cells lines were carried out for only those fractions of saponins with highly significant growth inhibition in MTT and NRU assay. Zf.Sa saponins showed prominent activity against all three cell lines (A3, B3 and C3). Microscopic examination revealed that the cells lost their shapes and were shrunken. It was a clear indication of nuclear fragmentation and chromatin condensation. It was very clear that the numbers of apoptotic cells were higher as compared to untreated cells (Control). The crude saponins isolated from leaves (Zl.Sa) and bark (Zb.Sa) were evaluated against MDA MB-468 breast cancer cells and were stained with DAPI. The treated cells showed similar results and revealed nuclear fragmentation and chromatin condensation. The saponins from bark and leaves showed no prominent activity against caco-2 cancer cell lines (Fig. 1).

## Discussion

Screening of herbal drugs may lead to discovery of new mutagenic agents which can be an alternative source to the costly anticancer chemotherapeutic agents. Due to low toxicity and less cost, some medicinal plants have attracted the attention as alternative cancer therapies [20]. The plants of genus Zanthoxylum have been traditionally used since ancient times and have been of much interest due to historical claims of anticancer properties. In general, several other species of the genus Zanthoxylum have previously been shown to possess cytotoxicity against various human cancer and tumor cell lines. Thus, the cytotoxic activity of the extracts of *Z. armatum*, observed in the present study, corresponds well with the cytotoxic potential of this genus [21]. An example of such evidence is of traditional Chinese medicine *Z. nitidum,* which has been reported to inhibit breast cancer cell lines [22].

MTT assay has been used to determine number of viable cells in an anticancer investigation [23]. Mitochondrial dehydrogenase (MD) produced by normal cells reduce MTT (Yellow) to formazan (blue). In dead cells MD lacks this activity and reduction of MTT not occurred [24]. MTT assay result showed that *Z. armatum*, the fruit extract showed significance inhibition of all the three cell lines in MTT assay. The bark and leaves saponins/extract of *Z. armatum* were significantly active against MDA MB-468 cells only while mild activity was observed against MCF-7 and Caco-2 cells. The neutral red uptake (NRU) assay also showed similar results for *Z. armatum* and inhibited the three cell lines in a manner observed in MTT assay.

Further studies were carried out on precipitated saponins fractions. Over 100 and 50 saponins from natural sources have been tested and found to be effective against different types of cancers. Due to this diversity in structure the saponins exert anticancer effect through variety of mechanisms and pathways. One of example is of steroidal saponins which induce apoptosis and cell cycle arrest of tumors [25]. Another report revealed that there is a growing interest in clinical use of saponins as chemotherapeutic agent. More than 400 reports are available about ability of saponins to induce apoptosis in treating cancer [26]. In one such study saponins compounds were experimented against colon cancer cell lines and it was found that saponins inhibited the tumor cells growth without altering the normal colon morphology [27]. Apoptosis is a programmed process of cell death. It occurs in pathological and physiological conditions. A defect in apoptotic pathways has an important role in carcinogenesis. In apoptosis, the chromatin condenses, the cells shrink, the DNA fragment and apoptotic bodies are formed. These all together are the characteristic of apoptosis [28]. It is reported that saponins exert its cytotoxic activity through apoptosis through signaling pathways to prevent the tumor [29]. The induction of apoptosis for the prevention of cancer is therefore desirable.

There are evidences available from the previous studies that indicates the cytotoxic potential of *Z. alatum* leaves. One such study was carried out against the Ehrlich ascites carcinoma in which the isolated compound Zanthonitirle showed the cytotoxic effect in dose dependent manner [30]. Another study also revealed the cytotoxic potential of lignans isolated *Z. alatum* against the pancreatic and lung carcinoma cell lines [31].

Here, we have studied the cytotoxic and apoptotic potential of the Zf.Sa, Zb.Sa and Zl.Sa on human cancer cell lines. The evidence already exist that the extracts of *Z. armatum* DC exert its cytotoxic potential by mechanisms involving apoptosis [3]. DAPI staining is most commonly used assay for observing the apoptosis at DNA level [19]. In this study saponins from *Z. armatum* fruit (Zf.Sa) induced morphological changes in apoptotic cells which were observed in DAPI staining. The confocal microscopy demonstrates that the treatment with saponins (Zf.Sa) resulted in apoptotic body formation, chromatin condensation and nuclear fragmentation. It clearly indicates the potential of saponins to induce apoptosis against cancer cell lines.

## Conclusion

In conclusion, the current study clearly demonstrates that the extract and crude saponins from fruit, bark and leaves of traditional medicinal plant *Zanthoxylum armatum* DC., has the potential to exert its cytotoxic effect on cancer cell lines isolated from human by a mechanism involving apoptosis. The overall finding demonstrate that this plant could be a potential source of new anticancer compounds for possible drug development against cancer.

## Abbreviations

DAPI: 4′, 6-diamidino-2-phenylindole
DMEM: Dulbecco’s modified Eagle medium
DMSO: Dimethylsulphoxide
FBS: Fetal bovine serum
MTT: Methyl-thiazolyl tertrazolium
NRU: Neutral red uptake
PBS: Phosphate buffer saline
Zb: Zanthoxylum bark
ZF: Zanthoxylum fruit
Zf.Sa: Zanthoxylum fruit saponins
Zl: Zanthoxylum leaves

## References

[1] Cragg GM, Newman DJ, Snader KM (1997). Natural products in drug discovery and development. J Nat Prod.

[2] Farnsworth NR, Soejarto D. Global importance of medicinal plants. Conserv Med Plants. 1991;26:25–51.

[3] Kharshiing EV (2012). Aqueous extracts of dried fruits of *Zanthoxylum armatum* DC.,(Rutaceae) induce cellular and nuclear damage coupled with inhibition of mitotic activity in vivo. Amer J Plant Sci.

[4] Özkan M (2013). Zanthoxylum Usambarense (Engl.) Kokwaro (Rutaceae) extracts inhibit the growth of the breast cancer cell lines MDA-MB-231 and MCF-7, but not the brain tumour cell line U251 in vitro. Phytother Res.

[5] Chopra R, Nayar S, Chopra I (2006). Glossary of Indian medicinal plants, national institute of science communication and information resources.

[6] Jain S, Jain M (1972). Antifungal studies on some indigenous volatile oils and their combinations. Plant Med.

[7] Dikshit A, Husain A. Antifungal action of some essential oils against animal pathogens. Fitoterapia. 1984;​55:171–76.

[8] Nair A, Nair GA, Joshua C (1982). Confirmation of structure of the flavonol glucoside tambuletin. Phytochemistry.

[9] Kumar S, Müller K (1999). Inhibition of keratinocyte growth by different Nepalese Zanthoxylum species. Phytother Res.

[10] Islam A, Sayeed A, Bhuiyan M, Mosaddik M, Islam M (2001). Astaq Mondal khan G: antimicrobial activity and cytotoxicity of *Zanthoxylum budrunga*. Fitoterapia.

[11] Motsei M, Lindsey K, Van Staden J, Jäger A (2003). Screening of traditionally used south African plants for antifungal activity against *Candida albicans*. J Ethnopharmacol.

[12] Banerjee H, Pal S, Adityachaudhury N (1989). Occurrence of rutaecarpine in *Zanthoxylum budrunga*. Plant Med.

[13] Chen I-S, Tsai I-W, Teng C-M, Chen J-J, Chang Y-L, Ko F-N, Lu MC, Pezzuto JM (1997). Pyranoquinoline alkaloids from *Zanthoxylum simulans*. Phytochemistry.

[14] Chen I-S, Wu S-J, Tsai I-L, Wu T-S, Pezzuto JM, Lu MC, Chai H, Suh N, Teng C-M (1994). Chemical and bioactive constituents from *Zanthoxylum simulans*. J Nat Prod.

[15] Dande PR, Talekar VS, Chakraborthy G (2010). Evaluation of crude saponins extract from leaves of *Sesbania sesban* (L.) Merr. For topical anti-inflammatory activity. Int J Res Pharm Sci.

[16] Borenfreund E, Babich H, Martin-Alguacil N (1988). Comparisons of two in vitro cytotoxicity assays-the neutral red (NR) and tetrazolium MTT tests. Toxicol in Vitro.

[17] Abdullah A-SH, Mohammed AS, Abdullah R, Mirghani MES, Al-Qubaisi M (2014). Cytotoxic effects of *Mangifera indica* L. kernel extract on human breast cancer (MCF-7 and MDA-MB-231 cell lines) and bioactive constituents in the crude extract. BMC Compl Alter Med.

[18] Borenfreund E, Puerner JA (1985). Toxicity determined in vitr o by morphological alterations and neutral red absorption. Toxicol Lett.

[19] Saha SK, Sikdar S, Mukherjee A, Bhadra K, Boujedaini N, Khuda-Bukhsh AR (2013). Ethanolic extract of the goldenseal, *Hydrastis canadensis*, has demonstrable chemopreventive effects on HeLa cells in vitro: drug–DNA interaction with calf thymus DNA as target. Environ Toxicol Pharmacol.

[20] Cassileth BR, Chapman CC (1996). Alternative and complementary cancer therapies. Cancer.

[21] Özkan M, Mutiso PB, Nahar L, Liu P, Brown S, Wang W, Sarker SD (2013). *Zanthoxylum usambarense* (Engl.) Kokwaro (Rutaceae) extracts inhibit the growth of the breast cancer cell lines MDA-MB-231 and MCF-7, but not the brain tumour cell line U251 in vitro. Phytother Res.

[22] ChengHui Y, MingJen C, ShiowJu L, ChengWei Y, HsunShuo C, IhSheng C (2009). Secondary metabolites and cytotoxic activities from the stem bark of *Zanthoxylum nitidum*. Chem Biodivers.

[23] Sylvester PW. Optimization of the tetrazolium dye (MTT) colorimetric assay for cellular growth and viability. In: Drug Design and Discovery. edn. New York: Springer. 2011;157–68. 10.1007/978-1-61779-012-6_9 21318905

[24] Lau C, Ho C, Kim C, Leung K, Fung K, Tse T, Chan H, Chow M (2004). Cytotoxic activities of *Coriolus versicolor* (Yunzhi) extract on human leukemia and lymphoma cells by induction of apoptosis. Life Sci.

[25] Man S, Gao W, Zhang Y, Huang L, Liu C (2010). Chemical study and medical application of saponins as anti-cancer agents. Fitoterapia.

[26] Thakur M, Melzig MF, Fuchs H, Weng A (2011). Chemistry and pharmacology of saponins: special focus on cytotoxic properties. Botanics Targets Therapy.

[27] MacDonald RS, Guo J, Copeland J, Browning JD, Sleper D, Rottinghaus GE, Berhow MA (2005). Environmental influences on isoflavones and saponins in soybeans and their role in colon cancer. J Nutr.

[28] Wong R (2011). Apoptosis in cancer: from pathogenesis to treatment. J Exp Clin Cancer Res.

[29] Han LT, Fang Y, Li MM, Yang HB, Huang F. The antitumor effects of triterpenoid saponins from the anemone flaccida and the underlying mechanism. Evidence-Based Compl Alter Med. 2013;2013. ​http://dx.doi.org/10.1155/2013/517931. 10.1155/2013/517931 PMC3804048 24191167

[30] Karmakar I, Haldar S, Chakraborty M, Dewanjee S, Haldar PK (2016). In vitro antioxidant and cytotoxic activity of Zanthonitrile isolated from *Zanthoxylum alatum*. J Appl Pharm Sci.

[31] Mukhija M, Dhar KL, Kalia AN (2014). Bioactive Lignans from *Zanthoxylum alatum* Roxb. Stem bark with cytotoxic potential. J Ethnopharmacol.

